# Crystal structure of 4-methyl-*N*-(4-methyl­benz­yl)benzene­sulfonamide

**DOI:** 10.1107/S2056989020000535

**Published:** 2020-01-17

**Authors:** Brock A. Stenfors, Richard J. Staples, Shannon M. Biros, Felix N. Ngassa

**Affiliations:** a Department of Chemistry, 1 Campus Dr., Grand Valley State University, Allendale, MI 49401, USA; b Center for Crystallographic Research, Michigan State University, Department of Chemistry and Chemical Biology, East Lansing, MI 48824, USA

**Keywords:** crystal structure, sulfonamide, N—H⋯O hydrogen bond, C—H⋯π inter­action

## Abstract

The title compound, C_15_H_17_NO_2_S, was synthesized *via* a substitution reaction between 4-methyl­benzyl­amine and *p*-toluene­sulfonyl chloride. In the crystal, N—H⋯O hydrogen bonds link the mol­ecules, forming ribbons running along the *b*-axis direction. One of the aromatic rings hosts two inter­molecular C—H⋯π inter­actions that link these hydrogen-bonded ribbons into a three-dimensional network.

## Abstract 2

The synthesis and crystal structure of the title aryl sulfonamide are described. In the crystal, N—H⋯O and C—H⋯π inter­actions link the mol­ecules, leading to the formation of a three-dimensional network structure.

## Chemical context   

Sulfonamides, commonly referred to as ‘sulfa drugs’, are a biologically significant class of drugs. Over 70 years since its discovery, the sulfonamide moiety is frequently used in modern medicine (Zhao *et al.*, 2016 ▸). First recognized as a class of anti­biotics in the 1930s, this class of drugs is used today to treat infectious diseases such as malaria, tuberculosis, HIV, and many more by targeting the di­hydro­pteroate synthase (DHPS) pathway (Dennis *et al.*, 2018 ▸). Sulfonamides also exhibit remarkable anti­tumor, anti­cancer, and anti­thyroid activities among others (Scozzafava *et al.*, 2003 ▸).
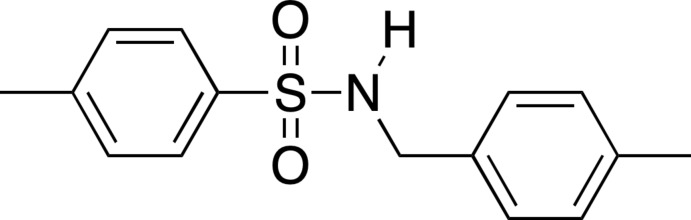


**Figure 1:**
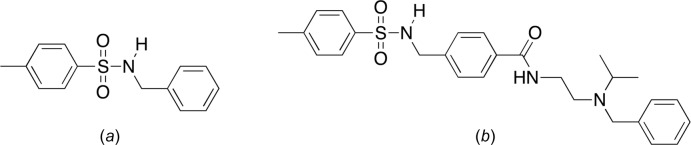
(*a*) *N*-benzyl-*p*-toluene sulfonamide (BTS) and (*b*) a kappa opioid receptor (KOR) antagonist containing the 4-methyl­benzyl­amine-4-methyl­benzene­sulfonamide moiety.

The title compound, 4-methyl­benzyl­amine-4-methyl­benzene­sulfonamide (I), is structurally similar to *N*-benzyl-*p*-toluene sulfonamide (BTS, Fig. 1 ▸). BTS is known to be a potent and specific inhibitor of the ATPase activity of skeletal myosin II subfragment 1 (S1) (Cheung *et al.*, 2002 ▸). The properties of BTS are significant in the study of muscle contraction (Pinniger *et al.*, 2005 ▸). In addition, the 4-methyl­benzyl­amine-4-methyl­benzene­sulfonamide moiety is found in a potent and selective kappa opioid receptor (KOR) antag­on­ist (Frankowski *et al.*, 2012 ▸; Fig. 1 ▸).

As therapeutic properties of sulfonamides continue to be discovered, it is important to synthesize these compounds efficiently. Sulfonamides are commonly synthesized by a mechanism analogous to the nucleophilic acyl-substitution reaction between an electrophile and a nucleophilic amine (Patel *et al.*, 2018 ▸). A review of the literature suggests that the most efficient method for synthesizing these compounds is by the sulfonyl­ation of amines using either sulfonyl halides or sulfonic acids as electrophiles (Yan *et al.*, 2007 ▸; De Luca & Giacomelli, 2008 ▸). The title compound was synthesized in di­chloro­methane using a sulfonyl chloride, in the presence of pyridine. The main purpose of pyridine is to act as a hydro­chloric acid scavenger. However, in our ongoing efforts to produce sulfonamides, we have recently discovered an environmentally benign and facile synthesis of aryl sulfonamides. This method uses aqueous potassium carbonate in tetra­hydro­furan. An increased rate of reaction and yield of sulfonamide compounds produced from a wide range of amines have been observed. We report here the synthesis of the title compound (I), as well as its mol­ecular and crystal structures.

## Structural commentary   

**Figure 2:**
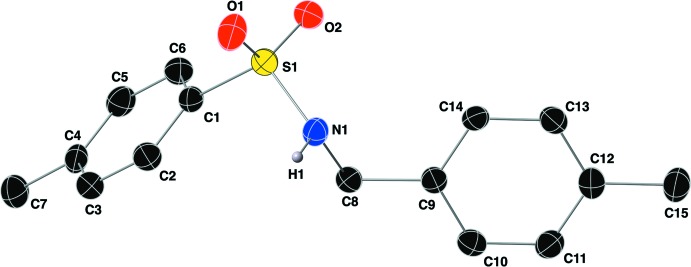
The mol­ecular structure of the title compound using standard CPK colors, showing the atom-labeling scheme. Aniosotropic displacement ellipsoids are shown at the 40% probability level.

The crystal structure of compound (I) was solved in the Sohnke space group *P*2_1_, with a Flack parameter of 0.06 (4). The mol­ecular structure is shown in Fig. 2 ▸ along with the atom-labeling scheme. The S=O bond lengths are 1.429 (2) and 1.424 (2) Å, with S1—N1 and S1—C1 bond lengths of 1.608 (2) and 1.764 (3) Å, respectively. The aryl groups of the sulfonamide are oriented gauche to one another with a C1—S1—N1—C8 torsion angle of 57.9 (2)°. The τ_4_ descriptor for fourfold coordination around the sulfur atom S1 is 0.94, indicating a slightly distorted tetra­hedral geometry of the sulfonamide group (where 0.00 = square-planar, 0.85 = trigonal–pyramidal, and 1.00 = tetra­hedral; Yang *et al.*, 2007 ▸). An intra­molecular C—H⋯O contact (Sutor, 1958 ▸,1962 ▸,1963 ▸; Table 1 ▸) is present between an aromatic C—H group and an O atom of the sulfonamide moiety in a *S*(5) motif (Table 1 ▸).

## Supra­molecular features   

**Figure 3:**
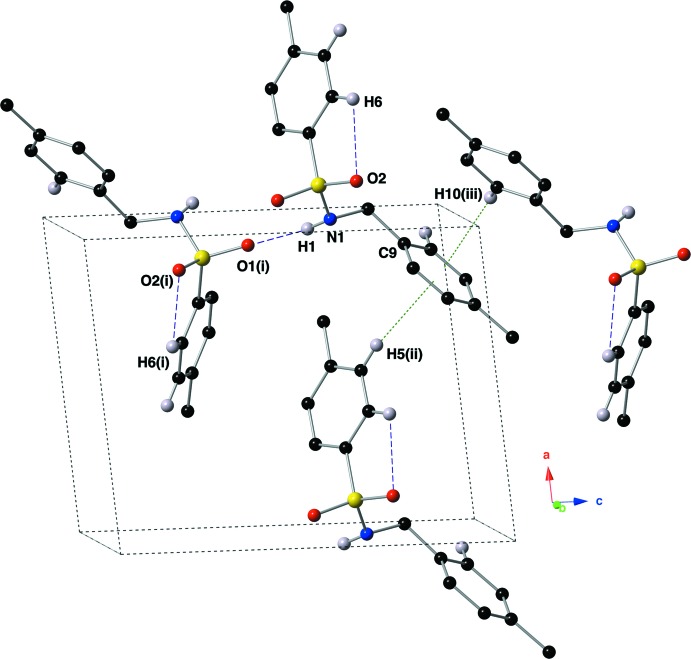
Depiction of the intra- and inter­molecular hydrogen bonds present in the structure of the title compound, using standard CPK colors with a ball-and-stick model. Hydrogen bonds and contacts are depicted with purple dashed lines, while C—H⋯π inter­actions are shown with green dotted lines. [Symmetry codes: (i) −*x*, −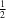 + *y*, 1 − *z*; (ii) 1 + *x*, *y*, *z*; (iii) −*x*, −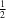 + *y*, 2 − *z*.]

**Figure 4:**
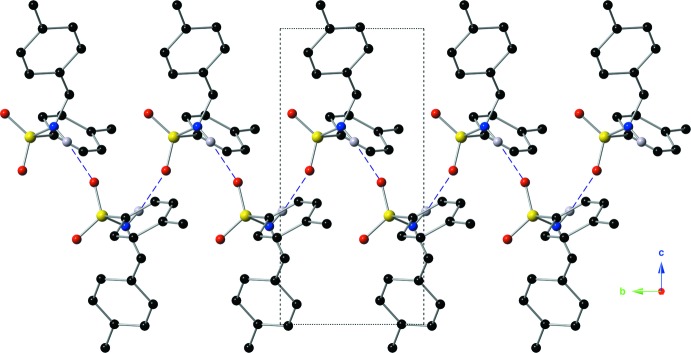
Depiction of the supra­molecular ribbons formed *via* inter­molecular N—H⋯O hydrogen bonds (purple dashed lines), as viewed down the *a* axis.

**Figure 5:**
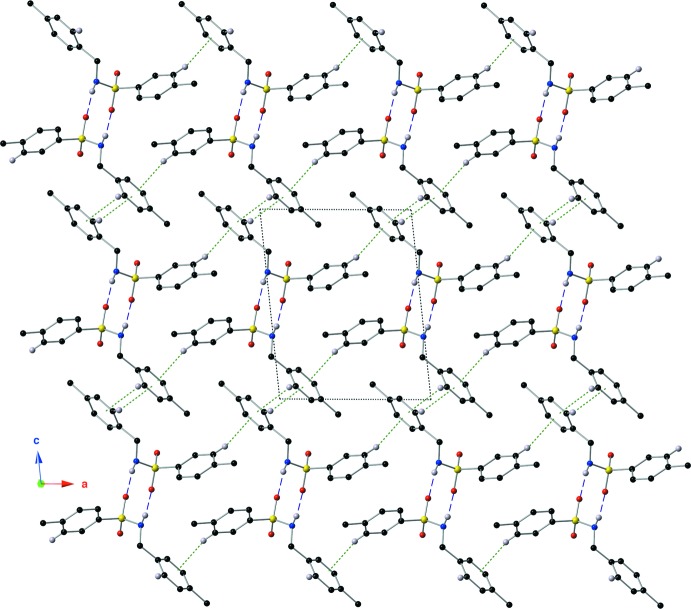
A view down the *b* axis of the crystal, showing the supra­molecular inter­actions. Hydrogen bonds and contacts and are shown with purple dashed lines, and C—H⋯π inter­actions are shown with green dotted lines. For clarity, only hydrogen atoms involved in a non-covalent inter­action are shown, and the intra­molecular hydrogen-bonding inter­actions have been omitted.

Mol­ecules of compound (I) exhibit both inter­molecular N—H⋯O hydrogen bonds and C—H⋯π inter­actions in the crystal structure (Fig. 3 ▸). The inter­molecular N1—H1⋯O1 hydrogen bond is of medium strength and links mol­ecules of title compound into ribbons that run parallel to the *b* axis (Table 1 ▸, Fig. 4 ▸). The C9–C14 ring hosts two C—H⋯π inter­actions that link the ribbons into an intricate three-dimensional network (Table 1 ▸, Fig. 5 ▸).

## Database survey   

The Cambridge Structural Database (CSD, Version 5.40, Aug 2019; Groom, *et al.*, 2016 ▸) contains 11 structures with the *N*-benzyl-*p*-toluene sulfonamide moiety. Included in this set is the structure of *N*-benzyl-*p*-toluene sulfonamide (BTS, Fig. 1 ▸). This structure has been deposited four times as PTSBZA–PTSBZA03 (Cameron, *et al.*, 1975 ▸; Yi-Ni, 2014 ▸; Bagchi *et al.*, 2014 ▸; Valerga & Puerta, 2016 ▸). Other structures that are closely related to the title compound are *N*-(2,4-di­meth­oxy­benz­yl)-4-methyl­benzene­sulfonamide (DERXAA; Hashmi *et al.*, 2006 ▸) and 2-(*p*-tosyl­amino­meth­yl)aniline (MILHIZ; Sanmartín *et al.*, 2007 ▸). All three crystal structures exhibit intra­molecular C—H⋯O hydrogen bonds, and MILHIZ is the only structure that does not show C—H⋯π inter­actions.

## Synthesis and crystallization   

The title compound was prepared by the dropwise addition of *p*-toluene­sulfonyl chloride (1.00 g, 5.25 mmol) to a stirring mixture of 4-methyl­benzyl­amine (0.75 ml, 5.90 mmol), pyridine (0.48 ml, 5.90 mmol) and 10 ml of degassed di­chloro­methane under a nitro­gen atmosphere. The reaction mixture was stirred at room temperature for 24 h under a nitro­gen atmosphere. The mixture was acidified with 5 *M* HCl and diluted with 15 ml of di­chloro­methane. The organic phase was washed with water. The aqueous layers were combined and back extracted with di­chloro­methane (10 ml). The combined organic layers were dried over anhydrous sodium sulfate and evaporated to dryness. The residue was dissolved in hot ethanol and filtered. The filtrate was transferred to a scintillation vial and, upon standing for 24 h, crystallized to afford pale-yellow crystals that were filtered from the mother liquor (42%; m.p. 376–378 K).

## Refinement   

Crystal data, data collection and structure refinement details are summarized in Table 2 ▸. All hydrogen atoms bonded to carbon atoms were placed in calculated positions and refined as riding: C—H = 0.95–1.00 Å with *U*_iso_(H) = 1.2*U*_eq_(C) for methyl­ene groups and aromatic hydrogen atoms, and *U*_iso_(H) = 1.5*U*_eq_(C) for methyl groups. The hydrogen atom bonded to the nitro­gen atom (H1) was located using electron-density difference maps. The N1—H1 bond distance was restrained using DFIX instructions in *SHELXL* (Sheldrick, 2015 ▸) at 0.88 Å to agree with the known value.

## Supplementary Material

Crystal structure: contains datablock(s) I. DOI: 10.1107/S2056989020000535/wm5537sup1.cif

Structure factors: contains datablock(s) I. DOI: 10.1107/S2056989020000535/wm5537Isup2.hkl

Supporting information file. DOI: 10.1107/S2056989020000535/wm5537Isup3.cml

CCDC reference: 1977684

Additional supporting information:  crystallographic information; 3D view; checkCIF report

## Figures and Tables

**Table 1:** Hydrogen-bond geometry (Å, °) *Cg* is the centroid of the C9–C14 ring

*D*—H⋯*A*	*D*—H	H⋯*A*	*D*⋯*A*	*D*—H⋯*A*
C6—H6⋯O2	0.95	2.51	2.890 (4)	104
N1—H1⋯O1^i^	0.86 (1)	2.03 (2)	2.889 (3)	170 (3)
C5—H5⋯*Cg* ^ii^	0.95	2.86	3.761 (3)	159
C10—H10⋯*Cg* ^iii^	0.95	2.89	3.564 (3)	129

Symmetry codes: (i) 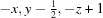; (ii) 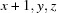; (iii) 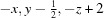.

**Table 2:** Experimental details

Crystal data	
Chemical formula	C_15_H_17_NO_2_S
*M*_r_	275.35
Crystal system, space group	Monoclinic, *P*2_1_
Temperature (K)	173
*a*, *b*, *c* (Å)	9.655 (2), 5.8820 (15), 12.180 (3)
β (°)	96.275 (3)
*V* (Å^3^)	687.5 (3)
*Z*	2
Radiation type	Mo *K*α
μ (mm^−1^)	0.23
Crystal size (mm)	0.49 × 0.22 × 0.16

Data collection	
Diffractometer	Bruker APEXII CCD
Absorption correction	Multi-scan (*SADABS*; Krause *et al.*, 2015 ▸)
*T*_min_, *T*_max_	0.474, 0.745
No. of measured, independent and observed [*I* > 2σ(*I*)] reflections	10794, 2811, 2619
*R*_int_	0.047
(sin θ/λ)_max_ (Å^−1^)	0.625

Refinement	
*R*[*F* ^2^ > 2σ(*F* ^2^)], *wR*(*F* ^2^), *S*	0.035, 0.092, 1.04
No. of reflections	2811
No. of parameters	178
No. of restraints	2
H-atom treatment	H atoms treated by a mixture of independent and constrained refinement
Δρ_max_, Δρ_min_ (e Å^−3^)	0.35, −0.21
Absolute structure	Flack *x* determined using 1114 quotients [(*I* ^+^)−(*I* ^−^)]/[(*I* ^+^)+(*I* ^−^)] (Parsons *et al.*, 2013 ▸)
Absolute structure parameter	0.06 (4)

Computer programs: *APEX2* and *SAINT* (Bruker, 2013 ▸), *SHELXS* (Sheldrick, 2008 ▸), *SHELXL2014* (Sheldrick, 2015 ▸), *OLEX2* (Dolomanov *et al.*, 2009 ▸; Bourhis *et al.*, 2015 ▸) and *CrystalMaker* (Palmer, 2007 ▸).

## supplementary crystallographic information

### Crystal data

**Table:**

C_15_H_17_NO_2_S	*F*(000) = 292
*M**_r_* = 275.35	*D*_x_ = 1.330 Mg m^−^^3^
Monoclinic, *P*2_1_	Mo *K*α radiation, λ = 0.71073 Å
*a* = 9.655 (2) Å	Cell parameters from 6778 reflections
*b* = 5.8820 (15) Å	θ = 2.6–26.4°
*c* = 12.180 (3) Å	µ = 0.23 mm^−^^1^
β = 96.275 (3)°	*T* = 173 K
*V* = 687.5 (3) Å^3^	Block, pale yellow
*Z* = 2	0.49 × 0.22 × 0.16 mm

### Data collection

**Table:**

Bruker APEXII CCD diffractometer	2619 reflections with *I* > 2σ(*I*)
φ and ω scans	*R*_int_ = 0.047
Absorption correction: multi-scan (*SADABS*; Krause *et al*., 2015)	θ_max_ = 26.4°, θ_min_ = 1.7°
*T*_min_ = 0.474, *T*_max_ = 0.745	*h* = −12→12
10794 measured reflections	*k* = −7→7
2811 independent reflections	*l* = −15→15

### Refinement

**Table:**

Refinement on *F*^2^	Hydrogen site location: mixed
Least-squares matrix: full	H atoms treated by a mixture of independent and constrained refinement
*R*[*F*^2^ > 2σ(*F*^2^)] = 0.035	*w* = 1/[σ^2^(*F*_o_^2^) + (0.0564*P*)^2^ + 0.0356*P*] where *P* = (*F*_o_^2^ + 2*F*_c_^2^)/3
*wR*(*F*^2^) = 0.092	(Δ/σ)_max_ < 0.001
*S* = 1.04	Δρ_max_ = 0.35 e Å^−^^3^
2811 reflections	Δρ_min_ = −0.21 e Å^−^^3^
178 parameters	Absolute structure: Flack *x* determined using 1114 quotients [(*I*^+^)-(*I*^-^)]/[(*I*^+^)+(*I*^-^)] (Parsons *et al.*, 2013)
2 restraints	Absolute structure parameter: 0.06 (4)
Primary atom site location: structure-invariant direct methods	

### Special details

**Table:**

Geometry. All esds (except the esd in the dihedral angle between two l.s. planes) are estimated using the full covariance matrix. The cell esds are taken into account individually in the estimation of esds in distances, angles and torsion angles; correlations between esds in cell parameters are only used when they are defined by crystal symmetry. An approximate (isotropic) treatment of cell esds is used for estimating esds involving l.s. planes.

### Fractional atomic coordinates and isotropic or equivalent isotropic displacement parameters (Å^2^)

**Table:**

	*x*	*y*	*z*	*U*_iso_*/*U*_eq_	
S1	0.12783 (6)	0.74015 (12)	0.63296 (5)	0.03224 (19)	
O1	0.0878 (2)	0.7954 (4)	0.51967 (17)	0.0460 (6)	
O2	0.1502 (2)	0.9174 (4)	0.71256 (18)	0.0414 (5)	
N1	0.0070 (2)	0.5761 (4)	0.66826 (19)	0.0323 (5)	
H1	−0.028 (3)	0.485 (5)	0.617 (2)	0.040 (9)*	
C1	0.2818 (3)	0.5768 (5)	0.6401 (2)	0.0307 (6)	
C2	0.2808 (3)	0.3720 (5)	0.5847 (2)	0.0344 (6)	
H2	0.1980	0.3194	0.5432	0.041*	
C3	0.4009 (3)	0.2447 (6)	0.5903 (2)	0.0352 (6)	
H3	0.4007	0.1047	0.5514	0.042*	
C4	0.5223 (3)	0.3174 (5)	0.6518 (2)	0.0323 (6)	
C5	0.5210 (3)	0.5225 (6)	0.7066 (2)	0.0381 (7)	
H5	0.6036	0.5745	0.7486	0.046*	
C6	0.4011 (3)	0.6544 (5)	0.7014 (2)	0.0358 (6)	
H6	0.4012	0.7955	0.7394	0.043*	
C7	0.6530 (3)	0.1762 (6)	0.6601 (3)	0.0434 (8)	
H7A	0.6287	0.0170	0.6440	0.065*	
H7B	0.7013	0.1882	0.7349	0.065*	
H7C	0.7141	0.2316	0.6067	0.065*	
C8	0.0272 (3)	0.4685 (5)	0.7768 (2)	0.0334 (6)	
H8A	0.1206	0.5097	0.8131	0.040*	
H8B	0.0248	0.3014	0.7671	0.040*	
C9	−0.0812 (3)	0.5358 (5)	0.8516 (2)	0.0298 (6)	
C10	−0.1137 (3)	0.3858 (5)	0.9318 (2)	0.0340 (6)	
H10	−0.0709	0.2403	0.9371	0.041*	
C11	−0.2081 (3)	0.4445 (5)	1.0049 (2)	0.0370 (6)	
H11	−0.2292	0.3385	1.0595	0.044*	
C12	−0.2721 (3)	0.6552 (5)	0.9995 (2)	0.0351 (6)	
C13	−0.2383 (3)	0.8054 (5)	0.9192 (2)	0.0343 (6)	
H13	−0.2810	0.9511	0.9139	0.041*	
C14	−0.1435 (2)	0.7477 (6)	0.8463 (2)	0.0321 (5)	
H14	−0.1212	0.8544	0.7924	0.038*	
C15	−0.3752 (3)	0.7190 (7)	1.0793 (2)	0.0451 (7)	
H15A	−0.4628	0.6372	1.0597	0.068*	
H15B	−0.3925	0.8831	1.0753	0.068*	
H15C	−0.3370	0.6779	1.1545	0.068*	

### Atomic displacement parameters (Å^2^)

**Table:**

	*U*^11^	*U*^22^	*U*^33^	*U*^12^	*U*^13^	*U*^23^
S1	0.0313 (3)	0.0305 (3)	0.0357 (3)	0.0031 (3)	0.0072 (2)	0.0024 (3)
O1	0.0453 (11)	0.0546 (16)	0.0393 (11)	0.0127 (10)	0.0100 (9)	0.0138 (10)
O2	0.0414 (11)	0.0331 (11)	0.0510 (13)	0.0007 (9)	0.0112 (9)	−0.0031 (10)
N1	0.0286 (11)	0.0358 (13)	0.0332 (12)	−0.0018 (10)	0.0060 (9)	−0.0034 (10)
C1	0.0282 (12)	0.0313 (14)	0.0334 (14)	0.0010 (10)	0.0076 (10)	0.0040 (12)
C2	0.0310 (13)	0.0343 (16)	0.0375 (15)	−0.0029 (11)	0.0023 (11)	−0.0027 (13)
C3	0.0380 (13)	0.0316 (13)	0.0369 (13)	0.0002 (14)	0.0076 (10)	−0.0011 (14)
C4	0.0288 (12)	0.0387 (15)	0.0310 (13)	0.0007 (11)	0.0103 (10)	0.0058 (11)
C5	0.0307 (13)	0.0430 (17)	0.0406 (16)	−0.0066 (12)	0.0041 (11)	−0.0015 (14)
C6	0.0357 (14)	0.0334 (14)	0.0387 (15)	−0.0054 (12)	0.0059 (11)	−0.0037 (12)
C7	0.0346 (14)	0.0469 (19)	0.0500 (17)	0.0068 (13)	0.0103 (12)	0.0019 (14)
C8	0.0310 (12)	0.0303 (14)	0.0397 (15)	0.0033 (11)	0.0079 (11)	0.0023 (12)
C9	0.0259 (12)	0.0294 (14)	0.0339 (14)	−0.0035 (10)	0.0030 (10)	−0.0044 (11)
C10	0.0332 (13)	0.0307 (14)	0.0375 (15)	0.0025 (11)	0.0013 (11)	0.0007 (12)
C11	0.0386 (14)	0.0362 (16)	0.0369 (15)	−0.0014 (12)	0.0072 (12)	0.0041 (12)
C12	0.0302 (13)	0.0417 (16)	0.0334 (14)	−0.0010 (12)	0.0037 (10)	−0.0062 (12)
C13	0.0285 (12)	0.0301 (15)	0.0443 (16)	−0.0001 (10)	0.0037 (11)	−0.0047 (12)
C14	0.0292 (11)	0.0284 (13)	0.0394 (13)	−0.0022 (13)	0.0068 (10)	0.0027 (15)
C15	0.0401 (14)	0.052 (2)	0.0458 (16)	0.0010 (16)	0.0152 (12)	−0.0031 (18)

### Geometric parameters (Å, º)

**Table:**

S1—O1	1.429 (2)	C7—H7C	0.9800
S1—O2	1.424 (2)	C8—H8A	0.9900
S1—N1	1.608 (2)	C8—H8B	0.9900
S1—C1	1.764 (3)	C8—C9	1.513 (4)
N1—H1	0.865 (13)	C9—C10	1.378 (4)
N1—C8	1.460 (4)	C9—C14	1.382 (4)
C1—C2	1.380 (4)	C10—H10	0.9500
C1—C6	1.380 (4)	C10—C11	1.385 (4)
C2—H2	0.9500	C11—H11	0.9500
C2—C3	1.375 (4)	C11—C12	1.383 (4)
C3—H3	0.9500	C12—C13	1.383 (4)
C3—C4	1.388 (4)	C12—C15	1.513 (4)
C4—C5	1.379 (4)	C13—H13	0.9500
C4—C7	1.504 (4)	C13—C14	1.385 (4)
C5—H5	0.9500	C14—H14	0.9500
C5—C6	1.389 (4)	C15—H15A	0.9800
C6—H6	0.9500	C15—H15B	0.9800
C7—H7A	0.9800	C15—H15C	0.9800
C7—H7B	0.9800		
			
O1—S1—N1	105.50 (13)	H7B—C7—H7C	109.5
O1—S1—C1	108.00 (12)	N1—C8—H8A	108.9
O2—S1—O1	119.71 (14)	N1—C8—H8B	108.9
O2—S1—N1	108.51 (12)	N1—C8—C9	113.5 (2)
O2—S1—C1	107.52 (13)	H8A—C8—H8B	107.7
N1—S1—C1	106.98 (13)	C9—C8—H8A	108.9
S1—N1—H1	115 (2)	C9—C8—H8B	108.9
C8—N1—S1	118.26 (18)	C10—C9—C8	119.1 (2)
C8—N1—H1	113 (2)	C10—C9—C14	118.6 (2)
C2—C1—S1	119.3 (2)	C14—C9—C8	122.3 (2)
C6—C1—S1	119.7 (2)	C9—C10—H10	119.6
C6—C1—C2	121.0 (3)	C9—C10—C11	120.9 (3)
C1—C2—H2	120.3	C11—C10—H10	119.6
C3—C2—C1	119.3 (3)	C10—C11—H11	119.5
C3—C2—H2	120.3	C12—C11—C10	120.9 (3)
C2—C3—H3	119.4	C12—C11—H11	119.5
C2—C3—C4	121.1 (3)	C11—C12—C15	120.9 (3)
C4—C3—H3	119.4	C13—C12—C11	117.9 (3)
C3—C4—C7	121.3 (3)	C13—C12—C15	121.2 (3)
C5—C4—C3	118.6 (3)	C12—C13—H13	119.3
C5—C4—C7	120.2 (3)	C12—C13—C14	121.3 (3)
C4—C5—H5	119.4	C14—C13—H13	119.3
C4—C5—C6	121.2 (3)	C9—C14—C13	120.4 (3)
C6—C5—H5	119.4	C9—C14—H14	119.8
C1—C6—C5	118.8 (3)	C13—C14—H14	119.8
C1—C6—H6	120.6	C12—C15—H15A	109.5
C5—C6—H6	120.6	C12—C15—H15B	109.5
C4—C7—H7A	109.5	C12—C15—H15C	109.5
C4—C7—H7B	109.5	H15A—C15—H15B	109.5
C4—C7—H7C	109.5	H15A—C15—H15C	109.5
H7A—C7—H7B	109.5	H15B—C15—H15C	109.5
H7A—C7—H7C	109.5		

### Hydrogen-bond geometry (Å, º)

*Cg* is the centroid of the C9–C14 ring

**Table:**

*D*—H···*A*	*D*—H	H···*A*	*D*···*A*	*D*—H···*A*
C6—H6···O2	0.95	2.51	2.890 (4)	104
N1—H1···O1^i^	0.86 (1)	2.03 (2)	2.889 (3)	170 (3)
C5—H5···*Cg*^ii^	0.95	2.86	3.761 (3)	159
C10—H10···*Cg*^iii^	0.95	2.89	3.564 (3)	129

Symmetry codes: (i) −*x*, *y*−1/2, −*z*+1; (ii) *x*+1, *y*, *z*; (iii) −*x*, *y*−1/2, −*z*+2.
